# The ERF transcription factor family in cassava: genome-wide characterization and expression analyses against drought stress

**DOI:** 10.1038/srep37379

**Published:** 2016-11-21

**Authors:** Wei Fan, Meirong Hai, Yunling Guo, Zehong Ding, Weiwei Tie, Xupo Ding, Yan Yan, Yunxie Wei, Yang Liu, Chunlai Wu, Haitao Shi, Kaimian Li, Wei Hu

**Affiliations:** 1 College of Resources and Environment, Yunnan Agricultural University, Kunming, 650201, China; 2 College of Agronomy and Biotechnology, Yunnan Agricultural University, Kunming, 650201, China; 3 Key Laboratory of Biology and Genetic Resources of Tropical Crops, Institute of Tropical Bioscience and Biotechnology, Chinese Academy of Tropical Agricultural Sciences, Haikou, 571101, China; 4 Hainan Key Laboratory for Sustainable Utilization of Tropical Bioresources, College of Agriculture, Hainan University, Haikou, 570228, China

## Abstract

Cassava (*Manihot esculenta*) shows strong tolerance to drought stress; however, the mechanisms underlying this tolerance are poorly understood. Ethylene response factor (ERF) family genes play a crucial role in plants responding to abiotic stress. Currently, less information is known regarding the ERF family in cassava. Herein, 147 ERF genes were characterized from cassava based on the complete genome data, which was further supported by phylogenetic relationship, gene structure, and conserved motif analyses. Transcriptome analysis suggested that most of the *MeERF* genes have similar expression profiles between W14 and Arg7 during organ development. Comparative expression profiles revealed that the function of *MeERF*s in drought tolerance may be differentiated in roots and leaves of different genotypes. W14 maintained strong tolerance by activating more *MeERF* genes in roots compared to Arg7 and SC124, whereas Arg7 and SC124 maintained drought tolerance by inducing more *MeERF* genes in leaves relative to W14. Expression analyses of the selected *MeERF* genes showed that most of them are significantly upregulated by osmotic and salt stresses, whereas slightly induced by cold stress. Taken together, this study identified candidate *MeERF* genes for genetic improvement of abiotic stress tolerance and provided new insights into ERF-mediated cassava tolerance to drought stress.

Plants are frequently challenged by unfavorable environmental factors, such as cold, drought, and high salinity in their life cycle, which severely affects their growth, development, yield, and quality^1^,^2^. On the other hand, Plants have developed various complicated mechanisms to cope with these abiotic stressors so that they can survive and complete their life cycle. Among them, the ABA-dependent and ABA-independent signal transduction pathways play important roles in plants responding to abiotic stress^3^. AREB and DREB transcription factors (TFs) are two major components involved in ABA-dependent and ABA-independent pathways, respectively^4^. The DREB subfamily and ERF subfamily form Ethylene Response Factor (ERF) family, which contains a single AP2 domain and lesser number of introns. Besides, the AP2, RAV, and Soloists families also contain the AP2 domain that is involved in DNA binding^5^,^6^.

In plants, accumulating evidence has shown that the transcriptional abundance of ERF family genes are regulated by various abiotic stressors and related signaling. A lot of ERF genes exhibited transcriptional changes when they are subjected to various stimuli, including cold, freezing, drought, salt, heat, ABA, and ethylene in various plant species^7^,^8^. Further genetic evidences also demonstrated the important roles of ERF family in plants’ responding to abiotic stress^9^,^10^. Additionally, biochemical studies have clarified the action of ERFs on the regulation of abiotic stress responses. The DREB subfamily genes activate multiple stress-responsive genes by interacting with the C-repeat/dehydration responsive element (CRT/DRE), which has a core motif of A/GCCGAC, in the promoters of target genes^11^,^12^. They regulate stress-responsive genes under various abiotic stresses, including low temperature^13^,^14^,^15^, heat^16^,^17^, drought^17^,^18^,^19^,^20^ and high salinity^18^. The ERF subfamily genes, which play more diverse functions than the DREB subfamily genes, was reported to induce or repress gene expression with external stimuli of ethylene, cytokinin and abiotic stresses, such as wounding, cold, high salinity, and drought by recognizing the GCC-box^21^,^22^,^23^,^24^,^25^. Together, these studies demonstrated that ERF family genes play a crucial role in abiotic stress and related signaling response, which can be used as excellent candidates for genetic breeding to enhance crop tolerances to abiotic stress.

To date, numerous ERF family members have been identified in several plant species, such as 122 ERF genes in *Arabidopsis thaliana*, 139 in *Oryza sativa*, 169 in *Populus trichocarpa*, 103 in *Cucumis sativus*, 90 in *Prunus mume*, 200 in *Musa acuminata*, and 243 in *Musa balbisiana*^5^,^6^. However, less information is known about the ERF family in cassava (*Manihot esculenta*), an important tropical crop. Cassava is the third most important crop after rice and maize in Africa, Asia, and Latin America^26^. Due to the prominent characteristic on starch storage, it provides staple food for over 600 million people worldwide and also supplies raw material for production of bioethanol and industrial starch^27^,^28^. Notably, cassava can effectively utilize light, heat and water resources, thus showing high resistance to drought and low-fertility environment^29^,^30^. However, it is less known for the mechanisms underlying cassava resistant to abiotic stress. Thus, investigation of its tolerance mechanisms to abiotic stress may provide effective clues for genetic improvement of stress tolerance. Previously, we finished the genome sequencing of wild ancestor and cultivated varieties of cassava, which provides an excellent chance for genome-wide analysis of cassava genes^31^. Considering the crucial roles of ERF genes in abiotic stress response, we perform systematic analysis of the cassava ERF family.

## Results

### Identification and phylogenetic analysis of cassava ERF family

To identify the ERF family genes from cassava, both Hidden Markov Model and BLAST searches were employed to search the cassava genome with Arabidopsis and rice ERF sequences as queries. Totally, 147 putative ERF members were identified from the cassava genome, which was further confirmed by conserved domain analysis showing that all the identified ERFs have the AP2/ERF domain. The 147 predicted ERF proteins vary from 132 (MeERF71) to 716 (MeERF12) amino acid in length, the relative molecular mass range from 14.7 (MeERF71) to 79.7 (MeERF12) kDa, and the isoelectric points are in the range of 4.51–9.8 (Supplementary Table S1).

**Figure 1:**
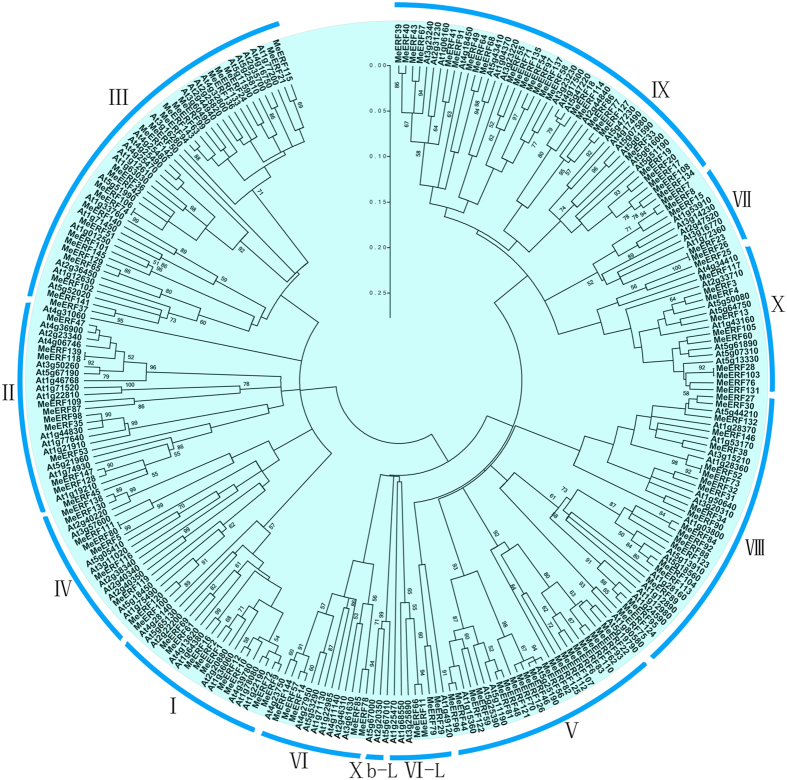
Phylogenetic analysis of ERFs from cassava and Arabidopsis. The Neighbor-joining (NJ) tree was constructed using Clustal X 2.0 and MEGA 5.0 softwares with the pair-wise deletion option. 1000 bootstrap replicates were used to assess tree reliability.

To characterize the evolutionary relationships between ERFs from cassava and other known ERFs from Arabidopsis, an Neighbor-Joining tree was constructed. As shown in Fig. 1, the 147 MeERFs could be divided to 12 groups, together with their orthologs from Arabidopsis. Phylogenetic analysis indicated that groups I, II, III and IV, groups V, VI, VI-L, VIII and Xb-L, and groups VII, IX and X were clustered, respectively, which was consistent with the classification of ERF family in Arabidopsis. Group III (22 AtERFs and 23 MeERFs) and Group IX (22 AtERFs and 23 MeERFs) accounted for the largest two groups among the 12 groups. Group VI and VI-L only contained five (MeERF14, MeERF57, MeERF78, MeERF85, MeERF144) and four (MeERF11, MeERF 29, MeERF 66, MeERF 79) members, respectively. According to the evolutionary relationship, some closely related orthologous ERFs appeared between cassava and Arabidopsis, implying that an ancestral set of ERFs existed before the divergence of cassava and Arabidopsis.

### Gene structure and conserved motifs of cassava ERFs

**Figure 2:**
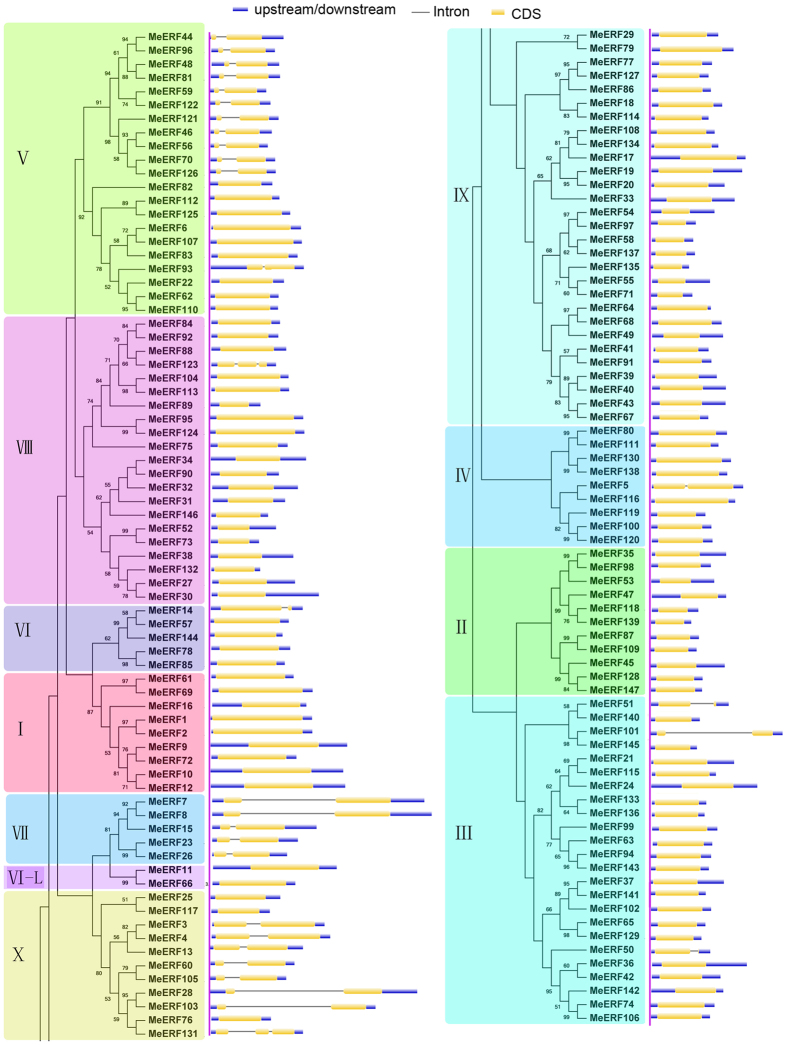
Gene structure analyses of cassava ERFs according to phylogenetic relationship. Exon-intron structure analyses were performed by GSDS database. The blue boxes, yellow boxes, and the black lines indicate upstream/downstream, exons, and introns, respectively.

To better understand the structural features of *MeERFs*, intron/exon structure was detected based on the evolutionary relationships (Fig. 2). The results displayed that the number of introns of *MeERFs* varied from 0–2. Among the 147 *MeERFs*, 116 *MeERF*s that almost distributed in all groups, except for group V, VII and X, were detected to have no intron. Besides, 29 *MeERFs* distributing in group V, VII and X contained 1 intron. *MeERF123* and *MeERF131* were found to have 2 introns. Generally, most of *MeERFs* in the same groups showed similar exon-intron feature, which supports their close evolutionary relationship and the classification of groups.

**Figure 3:**
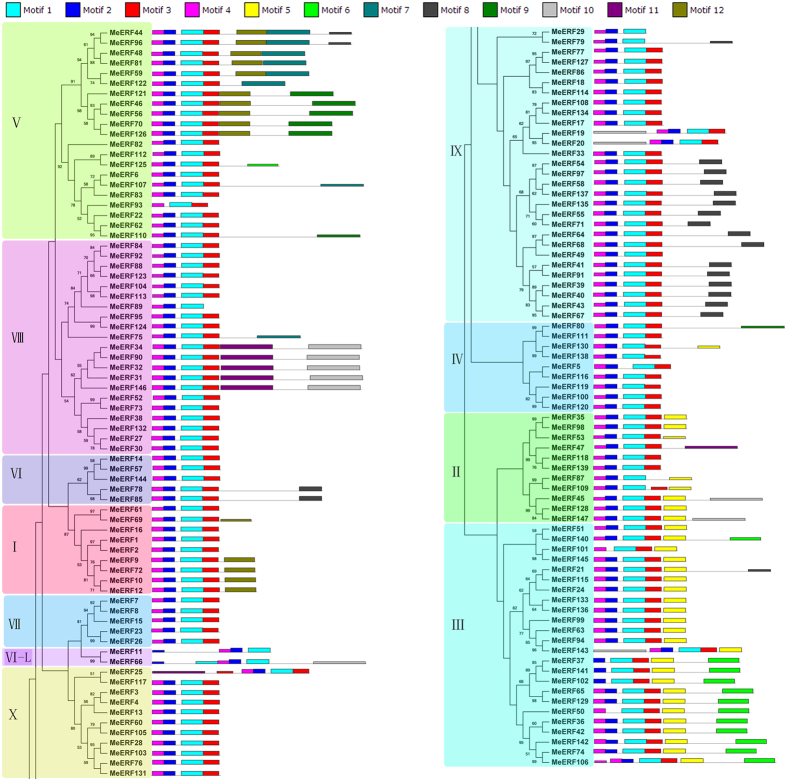
The conserved motifs of cassava ERFs according to phylogenetic relationship. All motifs were identified by MEME database with the complete amino acid sequences of cassava ERFs.

To investigate the structural and functional diversity of MeERF proteins, 12 conserved motifs in the MeERFs were captured by MEME software, and were annotated with InterProScan (Fig. 3; Supplementary Fig. S1). Motif 1 and Motif 2 were annotated as AP2/ERF domain, while the remaining 10 motifs had no annotation (Supplementary Table S2). All the identified MeERFs had AP2/ERF motif 1, and most of the MeERFs also showed motif 2, expect for MeERF50, -93, and -101. This indicates that all the MeERFs contain a typical feature of AP2/ERF domain. Notably, All the MeERFs, except for MeERF11, -29, -37, -50, -66, -79, -87, -89, -93, -101, -102, and -141, contained motifs 1 to 4, suggesting that these motifs play key roles in the function of ERF members. Motif 5 only presented in group II, III, and IV; motif 6 only appeared in group III and V; motif 7, 8, 9 and 12 mainly distributed in group V; and motif 10 and 11 mainly displayed in group VIII. These results showed that different groups harboring specific motifs could result in differentiation and diversity of gene function.

### Expression analyses of *MeERFs* in distinct organs of two cassava genotypes

**Figure 4:**
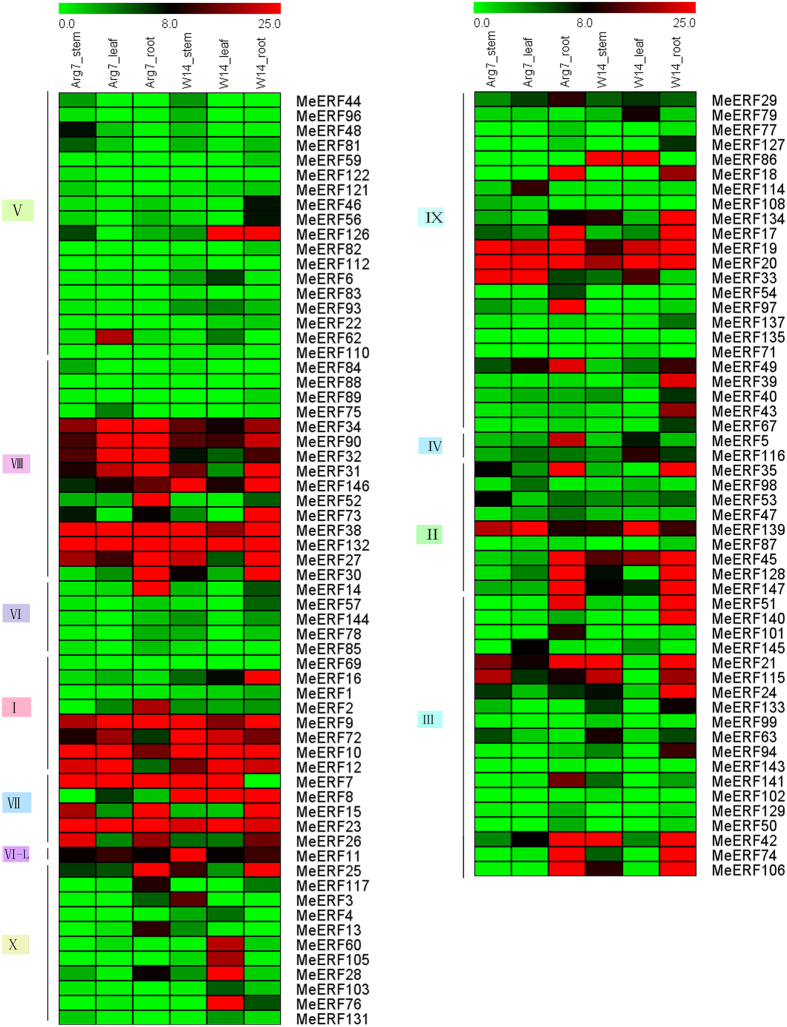
Expression profiles of cassava ERFs in tuberous roots, leaves, and stems of Arg7 and W14. The heat map was constructed according to the FPKM value of cassava ERFs. Changes in gene expression are shown in color as the scale.

To seek insights into the clues of *MeERFs* in cassava growth and development, the expression of *MeERFs* genes were examined in distinct organs, including tuberous roots, stems, and leaves between cultivated varieties (Arg7) and wild subspecies (W14). Transcriptome analysis showed a transcript abundance of 116 *MeERFs* in different organs, while the remaining 31 *MeERFs* were not detected in the RNA-seq libraries (Fig. 4; Supplementary Table S3).

For Arg7 variety, 20 (17.2%), 21 (18.1%), and 42 (36.2%) genes exhibited high transcriptional abundance (value > 10) in stem, leaf, and tuberous roots, respectively. Moreover, there were 14 *MeERFs (MeERF-34, -90, -32, -31, -38, -132, -27, -9, -10, -7, -23, -19, -20,* and *-139*) that had high expression levels (value > 10) in all the tested tissues. Additionally, 15 (12.9%), 23 (19.8%) and 17 (14.7%) genes did not show transcripts in stem, leaf, and tuberous roots, respectively.

For W14 subspecies, 27 (23.3%), 23 (19.8%), 44 (46.3%) genes had high transcriptional abundance (value > 10) in stem, leaf, and tuberous roots, respectively. Moreover, 13 *MeERFs (MeERF-90, -164, -38, -132, -9, -72, -10, -12, -8, -23, -19, -20, -11, -5, -34, -43, -17, -31*, and *-20*) showed high transcriptional levels (value > 10) in all the tested tissues. Additionally, 16 (13.8%), 19 (16.4%) and 15 (12.9%) genes did not show transcripts in stem, leaf, and tuberous roots, respectively.

Comparative analysis of expression profiles of *MeERFs* in different organs between Arg7 and W14 showed that 76 genes (65.5%) had transcripts abundance in all tissues of Arg7, while 83 genes (71.6%) in W14, suggesting the constitutive expression patterns for these genes. On the contrary, the failure to transcript detection might represent the distinct temporal or spatial expression patterns for the remaining *MeERFs*. Notably, 9 *MeERFs (MeERF-90, -38, -132, -9, -10, -23, -19, -20,* and *-139*) showed high expression abundance (value > 10) in all examined organs of Arg7 and W14, implying key roles for these genes in organs development. Overall, most of *MeERF* genes have similar expression profiles between W14 and Arg7 during organ development. Besides, Some *MeERF* genes exhibited differential expression profiles. Such as *MeERF-32, -15, -26* and *-33* had higher transcript abundance (value > 10) in stem of Arg7, whereas lower in stem of W14. On the contrary, *MeERF-146, -72, -8, -11, -25, -3, -86, -134, -45, -42,* and *-106* had higher transcriptional abundance (value > 10) in stem of W14, while lower in stem of W Arg7. This phenomenon was also observed in leaves and tuberous roots of Arg7 and W14.

### Expression profiles of *MeERFs* under drought stress in three cassava genotypes

**Figure 5:**
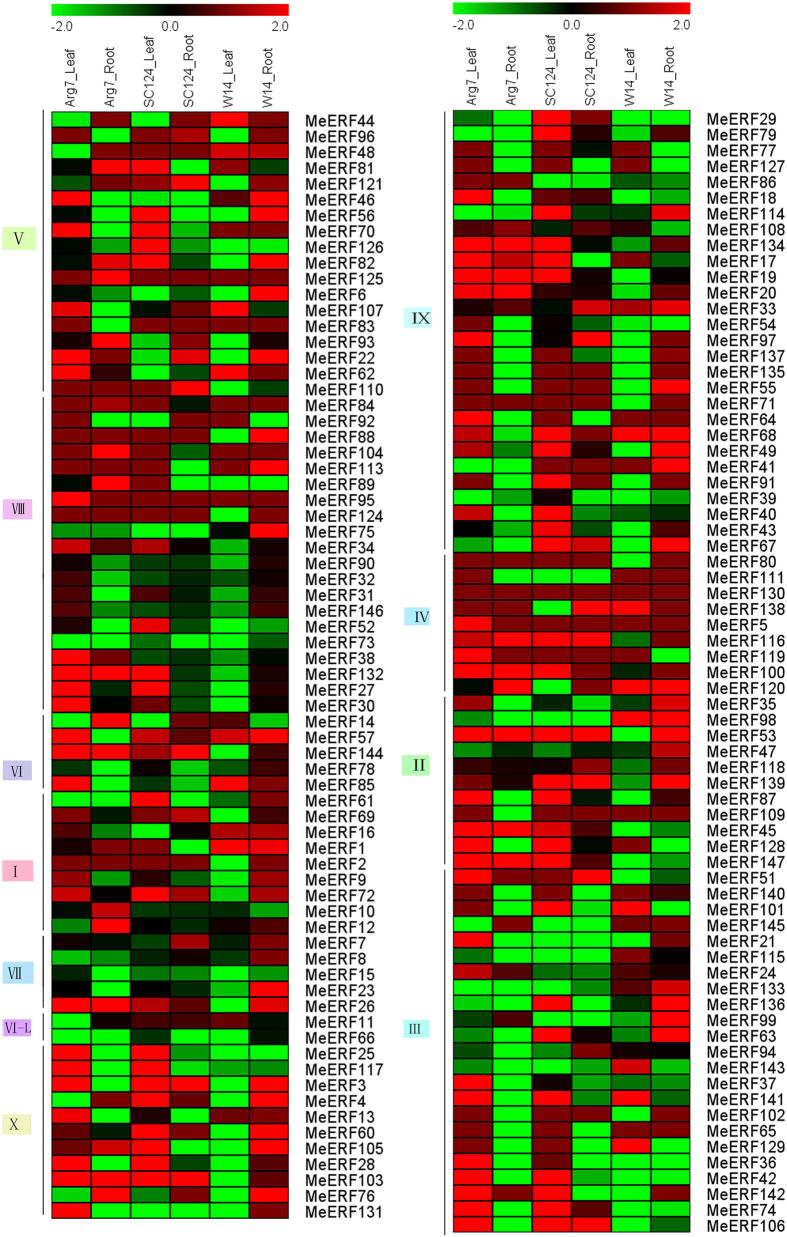
Expression profiles of cassava ERFs in response drought stress in Arg7, SC124, and W14 genotypes. Log2 based FPKM value was used to create the heat map. Changes in gene expression are shown in color as the scale.

To get some clues on the function of *MeERFs* in drought stress response, cassava seedlings of three genotypes were subjected to drought treatment and their leaves and roots tissues were sampled to perform transcriptome analysis (Fig. 5; Supplementary Table S4). According to the transcriptome data, 141 *MeERFs*, except for *MeERF50, -58, -59, -112, -122, -123* showed the corresponding expression data. Among the up-regulated genes (log2-based values > 1), 54 (38.3%) and 29 (20.6%) *MeERFs* were induced by drought in leaves and roots, respectively, in Arg7 variety; 51 (36.2%) and 22 (15.6%) *MeERFs* showed to be upregulated after drought treatment in leaves and roots, respectively, in South China 124 (SC124) variety; and 19 (13.5%) and 40 (28.4%) *MeERFs* were up-regulated by drought in leaves and roots, respectively, in W14 subspecies. These results indicated that the number of *MebZIPs* up-regulated by drought was greater in Arg7 and SC124 than those in W14. However, the number of drought*-*induced *MeERFs* in roots of W14 was significantly more than those in roots of Arg7 and SC124, whereas contrary in the leaves. Also, we found that 7 genes (*MeERF-46, -56, -75, -35, -98, -133, -136*) showed induction in roots of W14, whereas downregulation or no response in roots of Arg7 and SC124 after drought treatment. Six genes (*MeERF-70, -17, -40, -116, -100, and -128*) were upregulated in leaves of Arg7 and SC124, whereas downregulated or no response in leaves of W14 after drought treatment. These results suggested that the expression patterns of *MeERFs* responding to drought were similar between Arg7 and SC124, which differs from W14, and also suggested that the function of *MeERF*s underlying drought tolerance might be different between cultivated varieties and wild subspecies. In addition, some ERFs that had close relationship displayed different responses to drought stress, such as, *MeERF-21/-115*, *MebERF-39/-40*, *MeERF-3/-4*, and *MebERF-147/-128*. Together, the expression profiles of *MeERFs* responding to drought between cultivated varieties and wild subspecies will provide new clues to further investigation underlying mechanisms in cassava tolerance to drought.

### Expression profiles of *MeERFs* under osmotic, salt, and cold stresses

**Figure 6:**
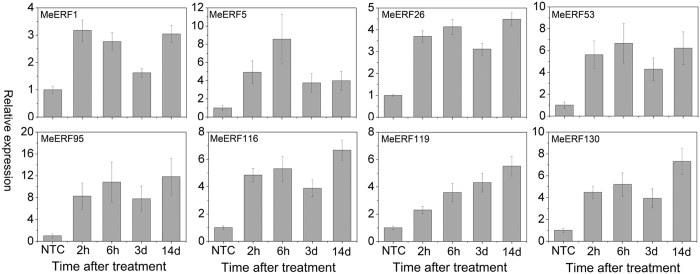
Expression profiles of cassava ERF genes in leaves of cassava in response to osmotic stress. The relative expression levels of each gene are presented as the mean fold changes between treated and control samples at each time point. NTC indicates no treatment controls (mean value = 1). Data are means ± SD of n = 3 biological replicates.

**Figure 7:**
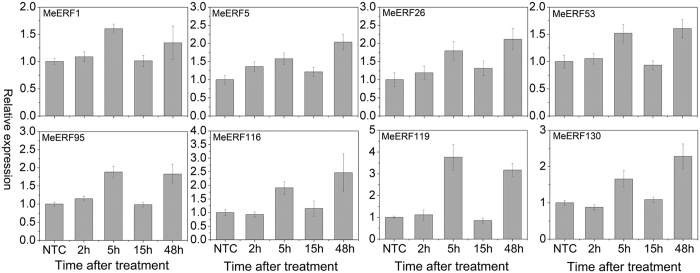
Expression profiles of cassava ERF genes in leaves of cassava in response to cold stress. The relative expression levels of each gene are presented as the mean fold changes between treated and control samples at each time point. NTC indicates no treatment controls (mean value = 1). Data are means ± SD of n = 3 biological replicates.

**Figure 8:**
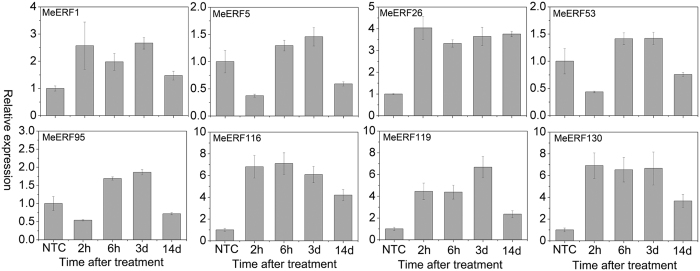
Expression profiles of cassava ERF genes in leaves of cassava in response to salt stress. The relative expression levels of each gene are presented as the mean fold changes between treated and control samples at each time point. NTC indicates no treatment controls (mean value = 1). Data are means ± SD of n = 3 biological replicates.

**Figure 9:**
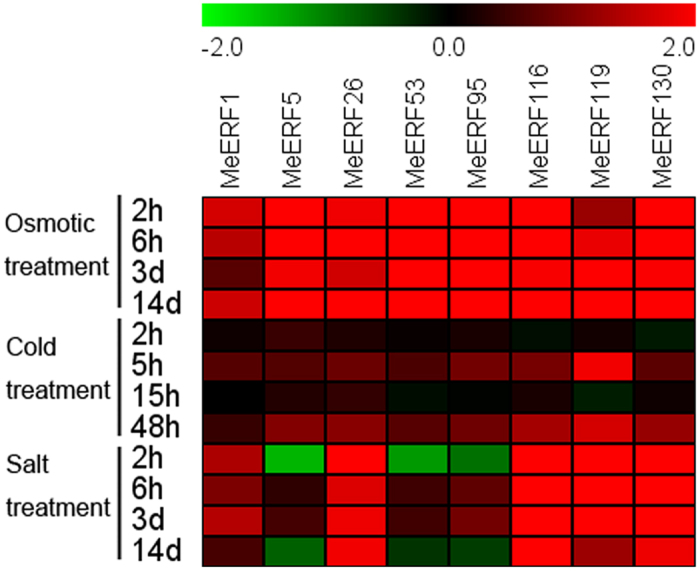
Expression profiles of cassava ERF genes in leaves under various stimuli. Log2 based values from three replicates of qRT-PCR data were used to create the heatmap. The scale represents the relative signal intensity values.

To examine the response of *MeERFs* to various environmental stresses at transcriptional levels, 8 *MeERFs (MeERF-1, -5, -26, -53, -95, -116, -119,* and *-130*) induced by drought based on transcriptome data in distinct cassava genotypes were chosen for further investigation of their expression patterns after osmotic, salt, and cold treatments. Under osmotic treatment, almost all the tested Me*ERFs* were significantly up-regulated (log2-based values > 1) during the whole treated time points (Fig. 6; Supplementary Table S5). Under cold treatment, the expression of all the tested *MeERFs* was slightly induced at 5 h and 48 h treatments (Fig. 7; Supplementary Table S5). Under salt treatment, *MeERF-26, -116, -119,* and *-130* were significantly induced (log2-based values > 1) during the treatment period, whereas *MeERF-1, -5, -53,* and *-95* did not show obvious trends, and were only up-regulated at several time points (Fig. 8; Supplementary Table S5). Together, these results indicated that most of the cassava *ERF* genes could be significantly upregulated by osmotic and salt stresses, whereas slightly affected by cold stress (Fig. 9; Supplementary Table S5).

### Validation of transcriptomic data

To validate the reliability of the RNA-seq data, we randomly selected 7 genes for quantitative real-time PCR (qRT-PCR) analysis. There was a good correlation (R = 0.77 for different organs; R = 0.78 for drought treatment) between RNA-seq data and the qRT-PCR results (Supplementary Fig. S2; Supplementary Fig. S3). These results indicated that the RNA-seq data could reflect the transcriptional changes.

## Discussion

Although cassava is an important crop, the research progress on cassava is still lagging compared to other crops. Physiologically and morphologically researches have characterized that cassava is a kind of crop that highly resistant to drought and low-fertility^29^,^30^,^31^,^32^,^33^. However, the molecular mechanisms underlying cassava responses to abiotic stress are poorly understood. ERF family has been widely reported as excellent candidates to improve plants tolerance in response to abiotic and biotic stresses, including cold, drought, salt, heat, fungal, and bacterial pathogens^17^,^21^. However, little is known for the *ERF* family in cassava.

Herein, 147 *ERF* family members were identified and characterized from cassava. Previous studies have identified 26 *ERFs* in *Picea sitchensis*, 32 in *Pinus taeda*, 67 in *Selaginella moellendorffii*, 90 in *Prunus mume*, 102 in *Sesamum Indicum*, 103 in *Cucumis sativus*, 122 in *Arabidopsis thaliana*, 139 in *Oryza sativa*, 169 in *Populus trichocarpa*, 200 in *Musa acuminata*, 243 in *Musa balbisiana*, and 248 in *Brassica rape*^5^,^6^,^34^,^35^. This suggested that *ERFs* in cassava had expanded in comparison to that in most species, while had shrunk relative to that in *Populus trichocarpa*, *Musa*, and *Brassica rape*. Evolutionary analysis indicated that the cassava ERFs was grouped into 12 subfamilies, which was further supported by analyses of gene structure and conserved motifs (Figs 1, 2 and 3). This classification was consistent with previous evolutionary analyses of ERFs in Arabidopsis and rice^5^. Gene structure analysis showed that most of the *MeERFs* showed no introns, except for the other 31 *MeERFs* genes containing one or two introns (Fig. 2). This phenomenon was similar to that of other species, such as Arabidopsis, rice, *Lotus corniculatus*, and *Sesamum Indicum*^5^,^35^,^36^,^37^, indicating that the evolution of gene structure of *ERF* family is relatively conservative compared to other transcription factor family, such as bZIPs and WRKY. Conserved motif analysis indicated that all the MeERFs contained a typical AP2/ERF domain (Fig. 3; Supplementary Table S2). Additionally, each subfamily had common motifs while some subfamilies contained the special motifs (Fig. 3). These features in conserved motifs of ERFs were also observed in other plant species^5^,^35^,^37^. Generally, most of MeERFs in the same subfamilies showed similar gene structure and conserved motifs.

ERF family transcription factors have been considered as important mediators of ethylene-mediated responses. Some ERF members can respond to abiotic stresses, such as drought and salt^10^,^21^, and overexpression of *ERF* genes resulted in increased tolerance to drought and salt stresses in transgenic plants^10^. Expression analyses of *ERF* family genes in poplar (*Populus trichocarpa*), soybean (*Glycine max*), and tomato (*Lycopersicon esculentum*) suggested that many *ERF* family genes showed transcriptional changes after high or low temperature treatments^38^,^39^. In this study, we found that many *MeERFs* could transcriptionally respond to drought stress in different genotypes (Fig. 5; Supplementary Table S4), implying possible function of these genes in response to drought stress in cassava. Interestingly, we noted that the number of *MeERFs* significantly induced by drought was greater in roots of W14 (28.4%) than that in roots of Arg7 (20.6%) and SC124 (15.6%), whereas less in leaves of W14 (13.5%) than that in leaves of Arg7 (38.3%) and SC124 (36.2%) (Fig. 5; Supplementary Table S4). Due to its deep root system, cassava can penetrate into deep soil layers and absorb water stored in soil^33^. Previous studies indicated that wild ancestor W14 was more tolerant to drought than cultivated varieties SC124 and Arg7^29^,^30^,^31^. Therefore, we hypothesized that the function of *MeERFs* in drought tolerance has been differentiated in the roots and leaves of different genotypes. High drought tolerance in W14 may be achieved by many members of the *MeERFs* in the roots to regulate absorption of water. Instead, Arg7 and SC124 are sensitive to drought, which requires more ERF genes expressed in leaves to well fit drought, thus remedying the deficiency of water uptake. Such a fine regulation patterns push us to further analysis the specific function of different members in different genotypes.

Further, 8 *MeERFs (MeERF-1, -5, -26, -53, -95, -116, -119,* and *-130*) upregulated by drought based on RNA-seq transcriptome data in different cassava genotypes were selected to examine their expression levels after osmotic, cold and salt treatments (Figs 6, 7, 8 and 9; Supplementary Table S5). The results indicated that most of the tested *MeERFs* were identified to be significantly up-regulated by osmotic and salt stresses. In Arabidopsis, the AtERF53 (the orthologous of MeERF1) is a drought-induced transcription factor that can regulate drought-responsive gene expressions by binding to the GCC box and/or dehydration-responsive element (DRE) in the promoter of downstream genes^36^,^40^. Overexpression of AtERF53 resulted in an unstable drought-tolerant phenotype^40^. Further studies showed that AtERF53 regulates drought and heat tolerances through interacting with RING domain ubiquitin E3 ligase for proteasome degradation^40^,^41^. Arabidopsis octadecanoid-responsive AP2/EFR-domain transcription factor ORA47 (the orthologous of MeERF53) is strongly induced by salt stress, wounding and MeJA^42^,^43^,^44^. Further studies suggested that ORA47 play a role in regulating a suite of genes related to biosynthesis and/or signaling transduction of phytohormone when plants are subjected to wounding and water stress^45^. Interestingly, MeERF-5, -116, -119, and -130 showed close phylogenetic relationship with 9 genes from Arabidopsis in group IV (Fig. 1). Accumulating evidence revealed that most of these homologous genes in Arabidopsis regulate salt and dehydration tolerances through activation of ABA-responsive network and stress-responsive genes^46^,^47^,^48^,^49^,^50^. These evidences suggested that these *MeERFs* might be positively involved in drought and salt tolerances of cassava. On the contrary, the expression of ERFs was slightly affected by cold stress, which could be explained by cassava is native to a warm habitat and is categorized as a cold-sensitive species^51^,^52^.

In conclusion, 147 *ERF* genes from cassava have been characterized based on evolutionary, conserved protein motif, and gene structure analyses, which will supply abundant information for functional characterization of *MeERF* genes. The expression profiles of *MeERFs* in distinct organs of two cassava genotypes indicated that *MeERF* members showed similar or differential expression patterns between Arg7 and W14, thus assisting in understanding the molecular basis for tissue development and function. Transcriptome analysis of three cassava genotypes responding to drought stress revealed that more *MeERFs* were activated in the roots of W14, whereas in leaves of Arg7 and SC124, which advances the understanding of functional differentiation of *MeERFs* in adapting to drought between drought-tolerant and drought-sensitive cassava genotypes. Expression of *MeERFs* under various abiotic treatments indicated the significant responses of *MeERFs* to osmotic and salt stresses. Together, these data will supply abundant information for functional characterization of *MeERF* genes and advance the understanding of *MeERF*-mediated cassava tolerance to abiotic stress.

## Methods

### Plant materials and treatments

The cassava accessions W14, SC124 and Arg7 were propagated by cutting. Segments cut from cassava stems were taken from mother plants, and cultured in pots filled with soil and vermiculite (1:1). The plants were grown from April to July in 2013 under an environmentally controlled growth room with a 16 h/35 °C day and 8 h/20 °C night regime, and a relative humidity of 70%. Ninety-day-old stems, 90-day-old leaves, and 150-day-old tuberous roots were sampled from Arg7 and W14 under normal conditions to study the expression levels of cassava *ERF* genes in distinct organs. To detect the transcriptional changes of cassava *ERF* genes in response to drought, leaves and roots were collected from Arg7, SC124 and W14, respectively, under drought conditions for 12 d. For osmotic, cold, or salt treatments, two month old seedlings of Arg7 were suffered from 200 mm mannitol for 14 days, low temperature (4 °C) for 48 h, and 300 mM NaCl for 14 days, respectively. Time point of sampling was determined while phenotypic changes reached on a moderate level of stress (Supplementary Fig. S4).

### Identification and evolutionary analyses

Protein sequences of ERFs from cassava, rice, and Arabidopsis were downloaded from Phytozome, RGAP, and UniPort databases, respectively^53^,^54^,^55^. To identify the cassava ERF gene family, a local hidden Markov Model-based search was established by using known ERFs from other plants to search cassava genome database^56^. Subsequently, Arabidopsis and rice ERFs were used as queries to check the predicted MeERFs by BLAST analysis. Finally, CDD and PFAM databases were employed to examine the predicted MeERFs^57^,^58^. In addition, a neighbor-joining tree was built by Clustal X 2.0 and MEGA 5.0 softwares with conserved domain of ERFs from cassava and Arabidopsis^59^,^60^.

### Protein properties and sequence analyses

The molecular weight and isoelectric points of MeERFs were predicted by proteomics server ExPASy^61^. The conserved motifs on MeERFs were identified by MEME program with following parameters: maximum motif number was 12, and the optimum motifs width was set at 10 to 50^62^. Further, all of the identified motifs were annotated using InterProScan^63^. Finally, gene structure of MeERFs was analyzed with the help of Gene structure Display Server Program^64^.

### Transcriptome analysis

Stems, leaves and tuberous roots of Arg7 and W14 growing on normal condition, and leaves and roots of SC124, Arg7, and W14 under normal conditions and drought treatment were used to isolated total RNA for transcriptome analysis by plant RNeasy extraction kit (TIANGEN, Beijing, China). Then, cDNA libraries of each sample were constructed with 3 μg of total RNA following the Illumina manufacture protocol, and sequenced by Illumina GAII. Data analysis was carried out by previously described^65^. The generated transcriptomic data has been submitted to NCBI as supplied in Supplementary Table S6.

### Quantitative real-time PCR analysis

According to the method mentioned above, the leaf samples of Arg7 treated with osmotic, cold, or salt stress under different time point were collected to isolate total RNA. First-strand cDNA was synthesized from 1 μg of total RNA using SuperScript reverse transcriptase (Takara). One microliter (100 ng μL^−1^) of cDNA in 10 μL solution systems was used for quantitative analysis of gene expression with SYBR Premix Ex Taq (Takara) on a Stratagene Mx3000P Real-Time PCR system. Agarose gel electrophoresis, melting curve, and sequencing analyses were performed to confirm the specificities of primer pairs. The primers of target genes were listed in Supplementary Tables S7 and S8. Each treated sample had a corresponding regularly-watered control at each time point. For each target gene, expression data were normalized with expression levels of β-tubulin gene (TUB) and elongation factors 1α gene (EF1) and calculated by the formula 2^−△△Ct^ 66,67.

## Additional Information

**How to cite this article**: Fan, W. *et al.* The ERF transcription factor family in cassava: genome-wide characterization and expression analyses against drought stress. *Sci. Rep.* **6**, 37379; doi: 10.1038/srep37379 (2016).

**Publisher’s note:** Springer Nature remains neutral with regard to jurisdictional claims in published maps and institutional affiliations.

## Supplementary Material

Supplementary Information

Supplementary Dataset 1

## References

[b1] Hu W., Huang C., Deng X., Zhou S. & Chen L. TaASR1, a transcription factor gene in wheat, confers drought stress tolerance in transgenic tobacco. Plant Cell Environ. 36, 1449–1464 (2013). 23356734 10.1111/pce.12074

[b2] Yang Q. S. *et al.* Comparative transcriptomics analysis reveals difference of key gene expression between banana and plantain in response to cold stress. BMC Genomics. 16, 446 (2015). 26059100 10.1186/s12864-015-1551-z PMC4461995

[b3] Agarwal P. K. & Jha B. Transcription factors in plants and ABA dependent and independent abiotic stress signaling. Biol Plantarum. 54, 201–212 (2010).

[b4] Zhu J. K. Salt and drought stress signal transduction in plants. Annu Rev Plant Biol. 53, 247–273 (2002). 12221975 10.1146/annurev.arplant.53.091401.143329 PMC3128348

[b5] Nakano T., Suzuki K., Fujimura T. & Shinshi H. Genome-wide analysis of the ERF gene family in Arabidopsis and rice. Plant Physiol. 140, 411–432 (2006). 16407444 10.1104/pp.105.073783 PMC1361313

[b6] Lakhwani D., Pandey A., Dhar Y. V., Bag S. K. & Trivedi P. K. Genome-wide analysis of the AP2/ERF family in *Musa* species reveals divergence and neofunctionalisation during evolution. Sci Rep. 6, 18878 (2016). 26733055 10.1038/srep18878 PMC4702079

[b7] Shu Y., Liu Y., Zhang J., Song L. & Guo C. Genome-Wide Analysis of the AP2/ERF Superfamily Genes and their Responses to Abiotic Stress in *Medicago truncatula*. Front Plant Sci. 6, 1247 (2016). 26834762 10.3389/fpls.2015.01247 PMC4717309

[b8] Chen L., Han J., Deng X., Tan S. & Li L. Expansion and stress responses of AP2/EREBP superfamily in *Brachypodium Distachyon*. Sci Rep. 6, 21623 (2016). 26869021 10.1038/srep21623 PMC4751504

[b9] Zhang H., Huang Z., Xie B., Chen Q. & Tian X. The ethylene-, jasmonate-, abscisic acid-and NaCl-responsive tomato transcription factor JERF1 modulates expression of GCC box-containing genes and salt tolerance in tobacco. Planta. 220, 262–270 (2004). 15300440 10.1007/s00425-004-1347-x

[b10] Hinz M., Wilson I. W., Yang J., Buerstenbinder K. & Llewellyn D. Arabidopsis RAP2.2: an ethylene response transcription factor that is important for hypoxia survival. Plant Physiol. 153, 757–772 (2010). 20357136 10.1104/pp.110.155077 PMC2879770

[b11] Huang G. T. *et al.* Signal transduction during cold, salt, and drought stresses in plants. Mol Biol Rep. 39, 969–987 (2012). 21573796 10.1007/s11033-011-0823-1

[b12] Yamaguchi-Shinozaki K. & Shinozaki K. A novel cis-acting element in an Arabidopsis gene is involved in responsiveness to drought, low-temperature, or high-salt stress. Plant Cell. 6, 251–264 (1994). 8148648 10.1105/tpc.6.2.251 PMC160431

[b13] Jaglo-Ottosen K. R. *et al.* Arabidopsis CBF1 overexpression induces COR genes and enhances freezing tolerance. Science. 280, 104–106 (1998). 9525853 10.1126/science.280.5360.104

[b14] Qin F. *et al.* Cloning and functional analysis of a novel DREB1/CBF transcription factor involved in cold-responsive gene expression in *Zea mays* L. Plant Cell Physiol. 45, 1042–1052 (2004). 15356330 10.1093/pcp/pch118

[b15] Kobayashi F. *et al.* Regulation by Vrn-1/Fr-1 chromosomal intervals of CBF-mediated Cor/Lea gene expression and freezing tolerance in common wheat. J Exp Bot. 56, 887–895 (2005). 15668223 10.1093/jxb/eri081

[b16] Novillo F., Alonso J. M., Ecker J. R. & Salinas J. CBF2/DREB1C is a negative regulator of CBF1/DREB1B and CBF3/DREB1A expression and plays a central role in stress tolerance in Arabidopsis. Proc Natl Acad Sci USA. 101, 3985–3990 (2004). 15004278 10.1073/pnas.0303029101 PMC374356

[b17] Mizoi J., Shinozaki K. & Yamaguchi-Shinozaki K. AP2/ERF family transcription factors in plant abiotic stress responses. Biochim Biophys Acta. 1819, 86–96 (2012). 21867785 10.1016/j.bbagrm.2011.08.004

[b18] Qin F. *et al.* Regulation and functional analysis of ZmDREB2A in response to drought and heat stresses in *Zea mays* L. Plant J. 50, 54–69 (2007). 17346263 10.1111/j.1365-313X.2007.03034.x

[b19] Sakuma Y. *et al.* Dual function of an Arabidopsis transcription factor DREB2A in water-stress-responsive and heat-stress-responsive gene expression. Proc Natl Acad Sci USA. 103, 18822–18827 (2006). 17030801 10.1073/pnas.0605639103 PMC1693746

[b20] Lim C. J. *et al.* Over-expression of the Arabidopsis DRE/CRT-binding transcription factor DREB2C enhances thermotolerance. Biochem Biophys Res Commun. 362, 431–436 (2007). 17716623 10.1016/j.bbrc.2007.08.007

[b21] Fujimoto S. Y. *et al.* Arabidopsis ethylene-responsive element binding factors act as transcriptional activators or repressors of GCC box-mediated gene expression. Plant Cell. 12, 393–404 (2000). 10715325 10.1105/tpc.12.3.393 PMC139839

[b22] Cutclife J. W., Hellmann E., Heyl A. & Rashotte A. M. CRFs form protein-protein interactions with each other and with members of the cytokinin signalling pathway in Arabidopsis via the CRF domain. J Exp Bot. 62, 4995–5002 (2011). 21705390 10.1093/jxb/err199 PMC3193008

[b23] Zwack P. J. *et al.* Vascular expression and C-terminal sequence divergence of cytokinin response factors in fowering plants. Plant Cell Physiol. 53, 1683–1695 (2012). 22864451 10.1093/pcp/pcs110

[b24] Allen M. D. *et al.* A novel mode of DNA recognition by a beta-sheet revealed by the solution structure of the GCC-box binding domain in complex with DNA. EMBO J. 17, 5484–5496 (1998). 9736626 10.1093/emboj/17.18.5484 PMC1170874

[b25] Rashotte A. M. & Goertzen L. R. The CRF domain defines cytokinin response factor proteins in plants. BMC Plant Biol 10, 74 (2010). 20420680 10.1186/1471-2229-10-74 PMC3095348

[b26] Oliveira E. J., Santana F. A., Oliveira L. A. & Santos V. S. Genetic parameters and prediction of genotypic values for root quality traits in cassava using REML/BLUP. Genet Mol Res. 13, 6683–6700 (2014). 25177949 10.4238/2014.August.28.13

[b27] Zidenga T., Leyva-Guerrero E., Moon H., Siritunga D. & Sayre R. Extending cassava root shelf life via reduction of reactive oxygen species production. Plant Physiol. 159, 1396–1407 (2012). 22711743 10.1104/pp.112.200345 PMC3425186

[b28] Perera P. I., Ordoñez C. A., Dedicova B. & Ortega P. E. Reprogramming of cassava (*Manihot esculenta*) microspores towards sporophytic development. AoB Plants. 6, plu022 (2014). 10.1093/aobpla/plu022 PMC4061485 24887001

[b29] Hu W., Yang H., Yan Y., Wei Y. & Tie W. Genome-wide characterization and analysis of bZIP transcription factor gene family related to abiotic stress in cassava. Sci Rep. 6, 22783 (2016). 26947924 10.1038/srep22783 PMC4780028

[b30] Wei Y., Shi H., Xia Z., Tie W. & Ding Z. Genome-Wide Identification and Expression Analysis of the WRKY Gene Family in Cassava. Front Plant Sci. 7, 25 (2016). 26904033 10.3389/fpls.2016.00025 PMC4742560

[b31] Wang W. *et al.* Cassava genome from a wild ancestor to cultivated varieties. Nat Commun. 5, 5110 (2014). 25300236 10.1038/ncomms6110 PMC4214410

[b32] El-Sharkawy M. A. International research on cassava photosynthesis, productivity, eco-physiology, and responses to environmental stresses in the tropics. Photosynthetica. 44, 481–512 (2006).

[b33] Okogbenin E. *et al.* Phenotypic approaches to drought in cassava: review. Front Physiol. 4, 93 (2013). 23717282 10.3389/fphys.2013.00093 PMC3650755

[b34] Song X., Li Y. & Hou X. Genome-wide analysis of the AP2/ERF transcription factor superfamily in Chinese cabbage (*Brassica rapa ssp. pekinensis*). BMC Genomics. 14, 573 (2013). 23972083 10.1186/1471-2164-14-573 PMC3765354

[b35] Dossa D., Wei X., Li D., Fonceka D. & Zhang Y. Insight into the AP2/ERF transcription factor superfamily in sesame and expression profiling of DREB subfamily under drought stress. BMC Plant Biol. 16, 171 (2016). 27475988 10.1186/s12870-016-0859-4 PMC4967514

[b36] Sakuma Y., Liu Q., Dubouzet J. G. & Abe H. DNA-binding specificity of the ERF/AP2 domain of Arabidopsis DREBs, transcription factors involved in dehydration- and cold-inducible gene expression. Biochem Biophys Res Commun. 290, 998–1009 (2002). 11798174 10.1006/bbrc.2001.6299

[b37] Sun Z. M., Zhou M. L., Xiao X. G., Tang Y. X. & Wu Y. M. Genome-wide analysis of AP2/ERF family genes from Lotus corniculatus shows LcERF054 enhances salt tolerance. Funct Integr Genomics. 14, 453–466 (2014). 24777608 10.1007/s10142-014-0372-5

[b38] Zhuang J., Cai B., Peng R. H., Zhu B. & Jin X. F. Genome-wide analysis of the AP2/ERF gene family in *Populus trichocarpa*. Biochem Biophys Res Commun. 371, 468–474 (2008). 18442469 10.1016/j.bbrc.2008.04.087

[b39] Sharma M., Kumar R., Solanke A., Sharma R. & Tyagi A. Identification, phylogeny, and transcript profiling of ERF family genes during development and abiotic stress treatments in tomato. Mol Genet Genomics. 284, 455–475 (2010). 20922546 10.1007/s00438-010-0580-1

[b40] Cheng M. C., Hsieh E. J., Chen J. H., Chen H. Y. & Lin T. P. Arabidopsis RGLG2, functioning as a RING E3 ligase, interacts with AtERF53 and negatively regulates the Plant drought stress response. Plant Physiol. 158, 363–375 (2012). 22095047 10.1104/pp.111.189738 PMC3252077

[b41] Hsieh E. J., Cheng M. C. & Lin T. P. Functional characterization of an abiotic stress-inducible transcription factor AtERF53 in *Arabidopsis thaliana*. Plant Mol Biol. 82, 223–237 (2013). 23625358 10.1007/s11103-013-0054-z

[b42] Wang Z. *et al.* Identification and characterization of COI1-dependent transcription factor genes involved in JA-mediated response to wounding in Arabidopsis plants. Plant Cell Rep. 27, 125–135 (2008). 17786451 10.1007/s00299-007-0410-z

[b43] Wasternack C. & Hause B. Jasmonates: biosynthesis, perception, signal transduction and action in plant stress response, growth and development. Ann Bot. 111, 1021–1058 (2013). 23558912 10.1093/aob/mct067 PMC3662512

[b44] Rehrig E. M., Appel H. M., Jones A. D. & Schultz J. C. Roles for jasmonate- and ethylene-induced transcription factors in the ability of Arabidopsis to responds differentially to damage caused by two insect herbivores. Front Plant Sci. 5, 407 (2014). 25191332 10.3389/fpls.2014.00407 PMC4137388

[b45] Chen H. Y. *et al.* ORA47 (octadecanoid-responsive AP2/ERF-domain transcription factor 47) regulates jasmonic acid and abscisic acid biosynthesis and signaling through binding to a novel cis-element. New Phytol. 211, 599–613 (2016). 26974851 10.1111/nph.13914

[b46] Song C., Je J., Hong J. K. & Lim C. O. Ectopic expression of an Arabidopsis dehydration-responsive element-binding factor DREB2C improves salt stress tolerance in crucifers. Plant Cell Rep. 33, 1239 (2014). 24737413 10.1007/s00299-014-1612-9

[b47] Li T., Wu X. Y., Li H., Song J. H. & Liu J. Y. A dual-function transcription factor, AtYY1, is a novel negative regulator of the Arabidopsis ABA response network. Mol Plant. 9, 650–661 (2016). 26961720 10.1016/j.molp.2016.02.010

[b48] Reis R. R. *et al.* Induced over-expression of AtDREB2A CA improves drought tolerance in sugarcane. Plant Sci. 221–222, 59–68 (2014). 10.1016/j.plantsci.2014.02.003 24656336

[b49] Lee S. Y., Boon N. J., Webb A. A. & Tanaka R. J. Synergistic Activation of RD29A via Integration of Salinity Stress and Abscisic Acid in Arabidopsis thaliana. Plant Cell Physiol. 57, 2147–2160 (2016). 10.1093/pcp/pcw132 PMC5434669 27497445

[b50] Lee S. J. *et al.* DREB2C interacts with ABF2, a bZIP protein regulating abscisic acid-responsive gene expression, and its overexpression affects abscisic acid sensitivity. Plant Physiol. 153, 716–727 (2010). 20395451 10.1104/pp.110.154617 PMC2879808

[b51] Huang L., Ye Z., Bell R. W. & Dell B. Boron nutrition and chilling tolerance of warm climate crop species. Ann Bot. 96, 755–767 (2005). 16033777 10.1093/aob/mci228 PMC4247042

[b52] An D., Yang J. & Zhang P. Transcriptome profiling of low temperature-treated cassava apical shoots dynamic responses of tropical plant to cold stress. BMC Genomics. 13, 64 (2012). 22321773 10.1186/1471-2164-13-64 PMC3339519

[b53] Prochnik S. *et al.* The Cassava genome: current progress, future directions. Trop Plant Biol. 5, 88–94 (2012). 22523606 10.1007/s12042-011-9088-z PMC3322327

[b54] The UniProt Consortium. UniProt: A hub for protein information. Nucleic Acids Res. 43, D204–D212 (2015). 25348405 10.1093/nar/gku989 PMC4384041

[b55] Kawahara Y. *et al.* Improvement of the *Oryza sativa* Nipponbare reference genome using next generation sequence and optical map data. Rice. 6, 4 (2013). 24280374 10.1186/1939-8433-6-4 PMC5395016

[b56] Finn R. D., Clements J. & Eddy S. R. HMMER web server: interactive sequence similarity searching. Nucleic Acids Res. 39, 29–37 (2011). 10.1093/nar/gkr367 PMC3125773 21593126

[b57] Marchler-Bauer A. *et al.* CDD: NCBI’s conserved domain database. Nucleic Acids Res. 43, D222–D226 (2015). 25414356 10.1093/nar/gku1221 PMC4383992

[b58] Finn R. D. *et al.* The Pfam protein families database. Nucleic Acids Res. 42, D222–D230 (2014). 24288371 10.1093/nar/gkt1223 PMC3965110

[b59] Larkin M. A. *et al.* Clustal W and Clustal X version 2.0. Bioinformatics. 23, 2947–2948 (2007). 17846036 10.1093/bioinformatics/btm404

[b60] Tamura K. *et al.* MEGA5: molecular evolutionary genetics analysis using maximum likelihood, evolutionary distance, and maximum parsimony methods. Mol Biol Evol. 28, 2731–2739 (2011). 21546353 10.1093/molbev/msr121 PMC3203626

[b61] Gasteiger E. *et al.* ExPASy: The proteomics server for in-depth protein knowledge and analysis. Nucleic Acids Res. 31, 3784–3788 (2003). 12824418 10.1093/nar/gkg563 PMC168970

[b62] Brown P. *et al.* MEME-LaB: Motif analysis in clusters. Bioinformatics. 29, 1696–1697 (2013). 23681125 10.1093/bioinformatics/btt248 PMC3694638

[b63] Mulder N. & Apweiler R. InterPro and InterProScan: Tools for protein sequence classification and comparison. Methods Mol. Biol. 396, 59–70 (2007). 18025686 10.1007/978-1-59745-515-2_5

[b64] Hu B. *et al.* GSDS 2.0: An upgraded gene feature visualization server. Bioinformatics. 31, 1296–1297 (2015). 25504850 10.1093/bioinformatics/btu817 PMC4393523

[b65] Hu W. *et al.* Genome-wide gene phylogeny of CIPK family in cassava and expression analysis of partial drought-induced genes. Front Plant Sci. 6, 914 (2015). 26579161 10.3389/fpls.2015.00914 PMC4626571

[b66] Livak K. J. & Schmittgen T. D. Analysis of relative gene expression data using real-time Quantitative PCR and the 2^–ΔΔCt^ method. Methods. 25, 402–408 (2001). 11846609 10.1006/meth.2001.1262

[b67] Salcedo A., Zambrana C. & Siritunga D. Comparative expression analysis of reference genes in field-grown cassava. Trop Plant Biol. 7, 53–64 (2014).

