# Supplementary material for: The ERF transcription factor family in cassava: genome-wide characterization and expression analyses against drought stress
Source: Sci Rep. 2016 Nov 21;6:37379. doi: 10.1038/srep37379 (PMC5116755; doi:10.1038/srep37379)
Supplement: Supplementary Information [file srep37379-s1.pdf]

Supplementary information for following article

The ERF transcription factor family in cassava: genome-wide characterization and expression analyses against drought stress

Wei Fan[1#], Meirong Hai[2#], Yunling Guo[3#], Zehong Ding[3], Weiwei Tie[3], Xupo Ding[3], Yan Yan[3], Yunxie Wei[4], Yang Liu[3], Chunlai Wu[3], Haitao Shi[4]*, Kaimian Li[3]*, Wei Hu[3]*

[1]College of Resources and Environment, Yunnan Agricultural University, Kunming, 650201, China

[2]College of Agriculture and Biotechnology, Yunnan Agricultural University, Kunming, 650201, China

[3]Key Laboratory of Biology and Genetic Resources of Tropical Crops, Institute of Tropical Bioscience and Biotechnology, Chinese Academy of Tropical Agricultural Sciences, Haikou, 571101, China

[4]Hainan Key Laboratory for Sustainable Utilization of Tropical Bioresources, College of Agriculture, Hainan University, Haikou, 570228, China

[#] These authors contributed equally to this work.

*Corresponding author: Haitao Shi (haitaoshi@hainu.edu.cn); Kaimian Li (likaimian@itbb.org.cn); Wei Hu (huwei2010916@126.com)

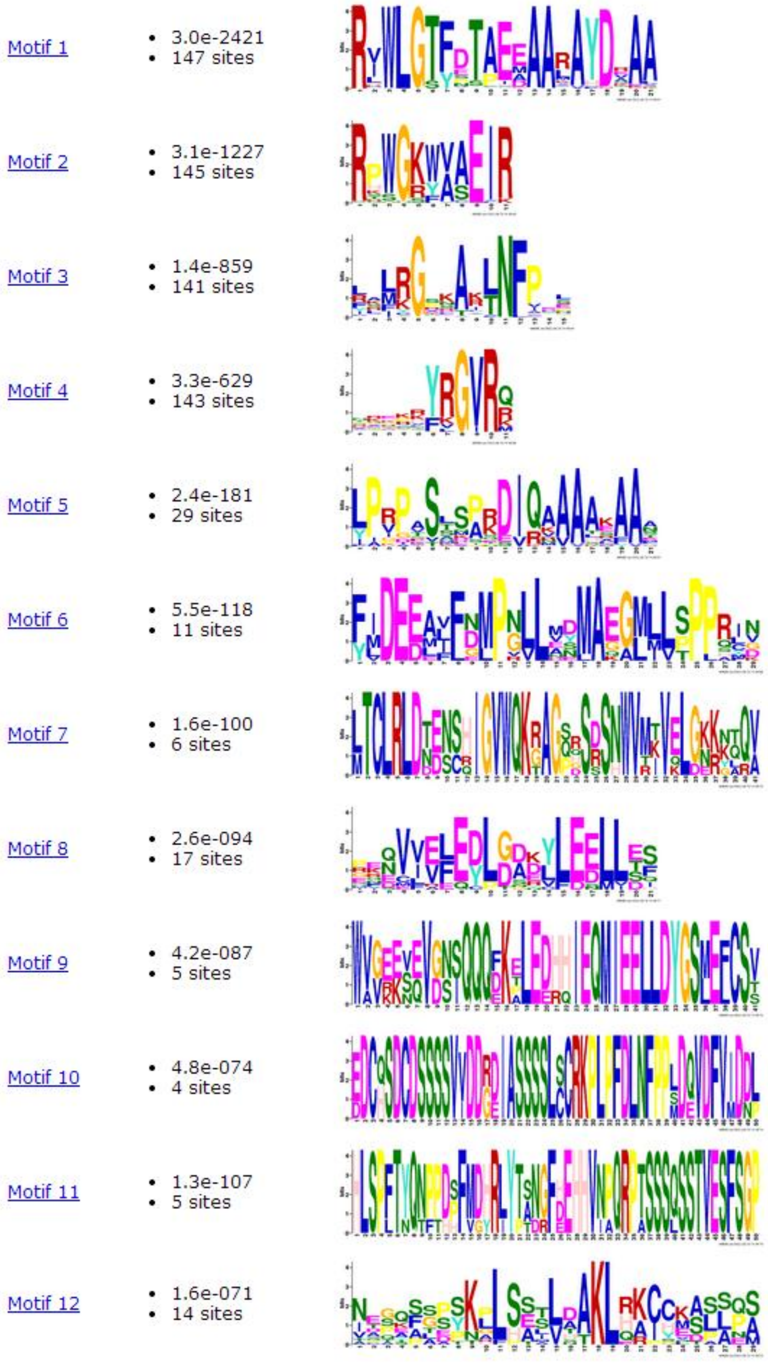

Supplementary Figure S1. The conserved motifs of cassava ERFs identified by MEME database with the complete amino acid sequences of cassava ERFs. 12 conserved motifs sharing high E-value were adopted.

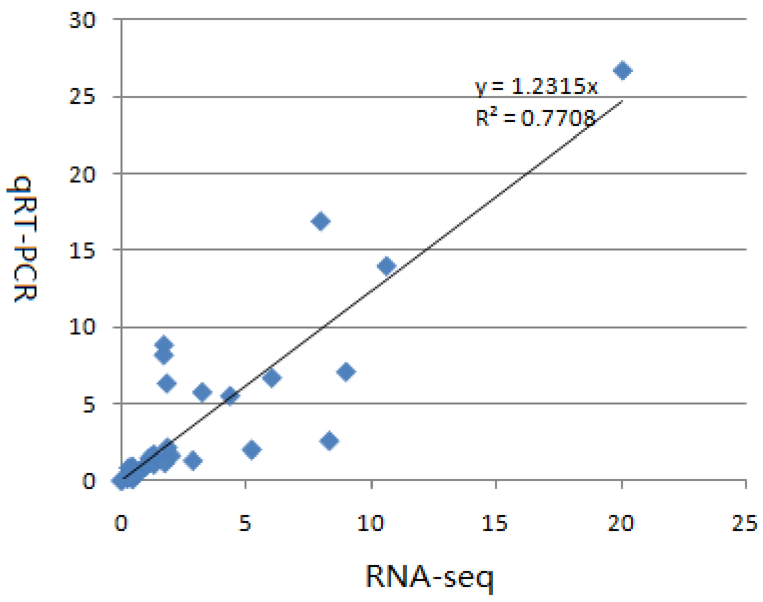

Supplementary Figure S2. Validation of the expression data in different organs of Arg7 and W14 by qRT-PCR analysis. Seven genes were randomly selected in the RNA-seq data for qRT-PCR analysis. Value of each RNA-seq expression data was plotted against that from qRT-PCR analysis.

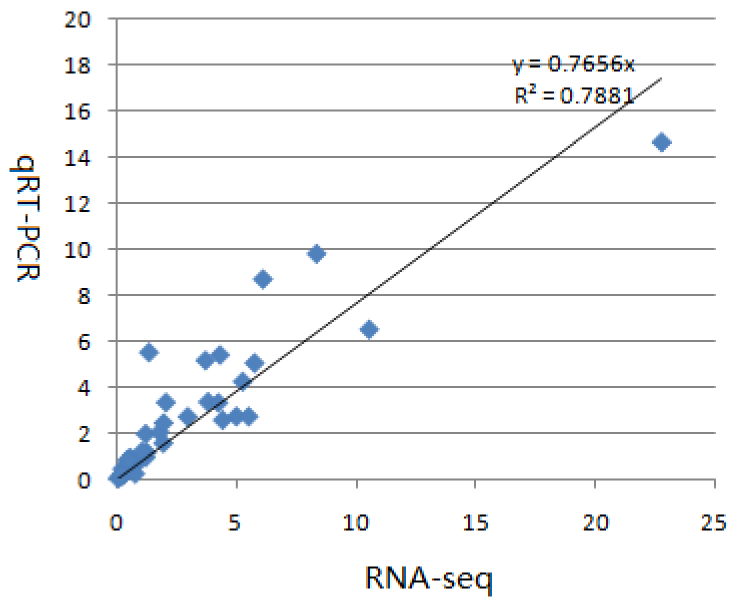

Supplementary Figure S3. Validation of the expression data related to drought treatment in Arg7, SC124, and W14 by qRT-PCR analysis. Seven genes were randomly selected in the RNA-seq data for qRT-PCR analysis. Value of each

RNA-seq expression data was plotted against that from qRT-PCR analysis.

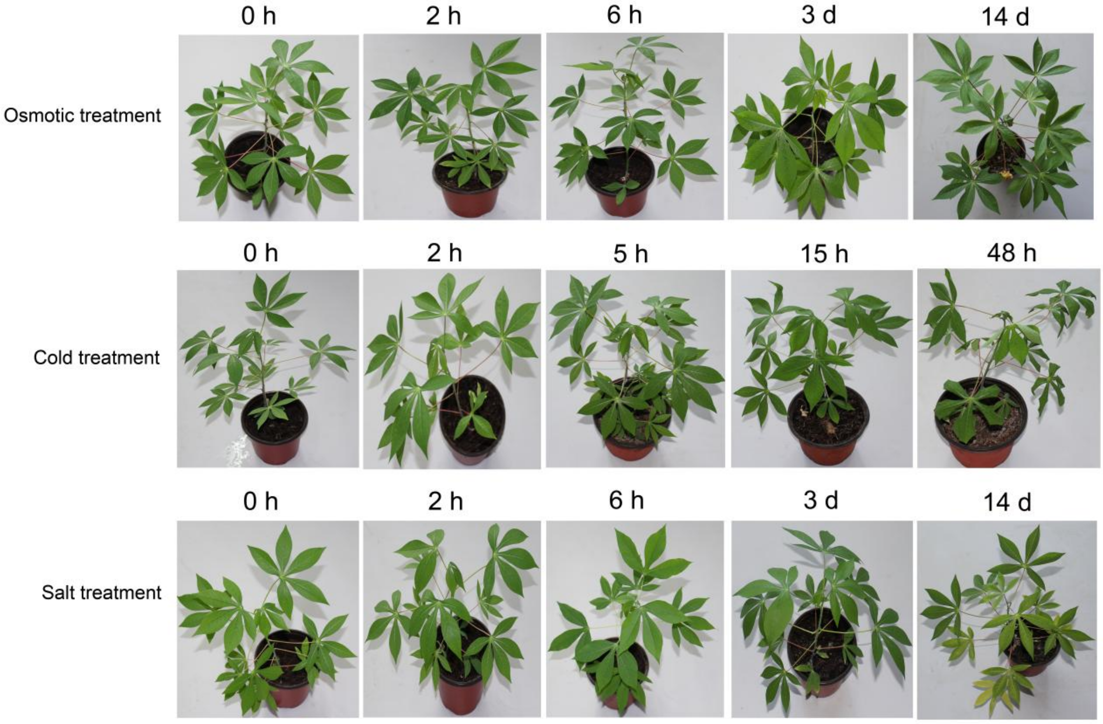

Supplementary Figure S4. Phenotypic changes of cassava treated with various stresses for different time. Time point of sampling was determined based on a moderate level of stress.
